# Covid-19 in kidney transplant recipients with immunosuppressive therapy

**DOI:** 10.22088/cjim.12.4.509

**Published:** 2022

**Authors:** Zahra Sheikhalipour, Masood Faghihdinevari, Hanieh Salehi-Pourmehr, Maryam Khameneh, Leila Vahedi

**Affiliations:** 1 Medical and Surgical Department, Nursing and Midwifery School, Organ Transplant Registry, Tabriz University of Medical Sciences, Iran; 2 Liver and Gastrointestinal Diseases Research Center, Organ Transplant Registry, Tabriz University of Medical Sciences, Tabriz, Iran; 3 Research Center for Evidence-Based Medicine, Iranian EBM Centre: A Joanna Briggs Institute (JBI) Center of Excellence, Tabriz University of Medical Sciences, Tabriz, Iran; 4 Student Research Committee, Islamic Azad University of Tabriz, Tabriz, Iran

**Keywords:** COVID- 19, Kidney transplant, Immunosuppressant, Review

## Abstract

**Background:**

Since the outbreak of COVID-19, various treatments have been frequently reported for patients infected with this virus, especially in transplant patients/recipients. Objective: Investigating of kidney transplant patients under immunosuppressive therapy infected with COVID-19 can pave the way to understanding, handling, and treatment of COVID-19.

**Methods:**

We had a brief review of the literature on immunosuppressive therapy in kidney transplants infected with COVID-19. This was based on the PubMed Database with keywords “kidney, transplant, COVID-19, and immunosuppress” after hospitalization of kidney transplantation infected with COVID-19. He had already been recorded in the Organ Transplant Registry (ID≠ 64510) of Tabriz University of Medical Sciences /Iran.

**Results:**

We reported the clinical course of a 45-year-old man with a history of kidney transplantation and immunotherapy who was infected with COVID-19 with respiratory infections and positive RT-PCR (Real-time polymerase chain reaction). He was treated with hydroxychloroquine, Kaletra, CellCept, and prednisolone for 5 days, and finally discharged from the hospital. In addition, reviewing of 47 papers with 851 samples showed that immunosuppressant medications alone could be a therapeutic choice in kidney transplants infected with COVID-19 with careful management.

**Conclusion:**

Patients with organ transplantation infected with COVID-19 may show different clinical signs, clinical course, and prognosis due to underlying diseases and the use of immunosuppressant medications. It might be best to continue taking the immunosuppressant medications but modify them based on the patients' conditions such as clinical symptoms, laboratory results, paraclinical examinations.

COVID-19, the first reported case in China's Wuhan region in late December 2019, has spread rapidly around the world and has become a pandemic disease (1, 2). The first case of COVID-19 in Iran was reported in February 2020, when all people, such as organ transplant patients, were exposed to the virus that caused COVID-19 (1, 3). COVID-19 infection causes variable clinical manifestations and outcomes (4, 5). However, it is possible that the clinical symptoms, clinical course, treatment, and prognosis in transplant recipients infected with COVID-19 may be different from other patients due to the presence of underlying diseases and the use of immunosuppressive drugs (4). Assessment immunosuppressant in transplant recipients infected with COVID‐19 is difficult because of the necessity to prevent graft rejection and excessive viral replication (5). Therefore, infection diagnosis, patient management and therapeutic management, especially safety-immunosuppressant regimen remain a challenge due to data shortages (6).

Following COVID-19 infection in a kidney transplant recipient who has already been registered in the Organ Transplant Registry (ID: 64510) of Tabriz University of Medical Sciences /Iran, studies were surveyed related to kidney transplant receipts under immunosuppressive therapy infected with COVID-19. In addition, we present the clinical course, diagnosis, treatment and outcome in this patient. Despite having a series of risk factors associated with poor prognosis, he had a relatively mild clinical course and was discharged from hospital after recovery. 

## Methods

We had a review on the literature associated with kidney transplant receipts under immunosuppressive therapy and infected with COVID-19 after hospitalization of a kidney transplantation in the section of care for organ transplant recipients infected with COVID-19. This patient has already been recorded in the Organ Transplant Registry Center (ID≠ 64510) of Tabriz University of Medical Sciences/Iran. Searching in the PubMed database with keywords such as “kidney, transplant, COVID-19, and immunosuppress” has been performed without time and language limitation. Letters, case reports, and case series were included in the study. The Preferred Reporting Items for Systematic Reviews (PRISMA) checklist and PICO include population, kidney transplants infected with COVID-19, intervention: consumption of immunosuppressant, comparison: no comparison and outcome: drug effects and COVID-19 infections. Titles and abstracts were reviewed and related articles were selected by authors. 

Then, the first author’s name, location of study, sample size, risk factors, treatment outcome, mortality rate, renal side effects, recommendations about immunosuppressant therapy were rigorously extracted by two researchers (V.L and S.Z) independently from the eligible studies. If there was a disagreement, the two researchers rechecked the original data of the included studies and had a discussion to reach to an agreement; then, the disagreements were adjudicated by the third researcher. 

**Ethics approval and consent to participate:** This study was approved by the Ethics Committee for Research of Tabriz/Iran University of Medical Sciences (decree number: TBZMED.REC.2020.423) and was performed in accordance with the ethical standards as laid down in the 1964 Declaration of Helsinki and its later amendments or comparable ethical standards. The information of patients and review literatures were gathered after the approval by the Deputy for Research of Tabriz University of Medical Sciences, gaining the ethical code, and obtaining permission from the patient and the Heads of Imam Reza Hospital and Transplant Registry (ID≠ 64510). The written consent was obtained from the patient before starting the report. Informed consent, and written informed consent were obtained from legal guardians. Verbal informed consent was obtained prior to the interview. The participant has consented to the submission of the case report to the journal. Patients signed informed consent regarding publishing their data.

## Results

**Figure 1:**
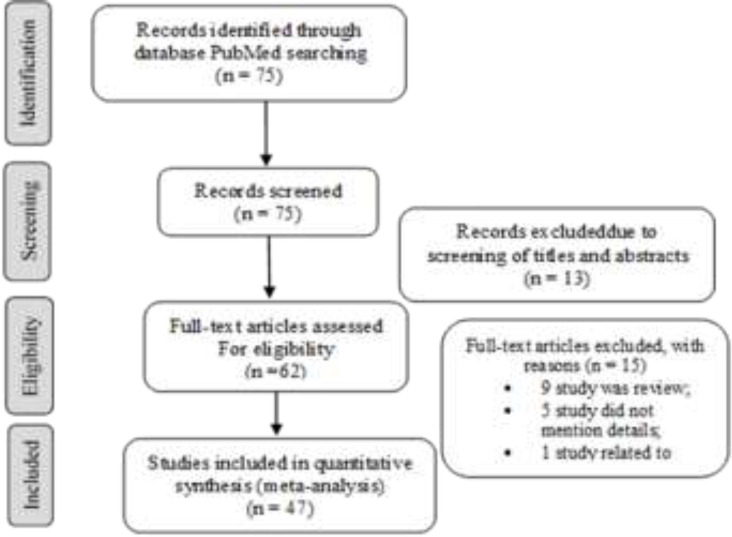
Flow diagram of studies for inclusion in the systematic review and meta-analysis

A total of 74 studies obtained from PubMed based on the inclusion criteria, 48 papers (3, 4, 7-52) with 851 samples were enrolled in our study. Twenty-six studies (12 studies due to irrelevance and 14 studies due to review or not to mention the necessary items) were excluded. A flowchart of the study selection process is presented in figure 1. 

The highest number of studies were related to Italy, China, and the USA, and the highest sample sizes were related to the USA, Italy, and China, respectively. The characteristics of the studies are shown in table1. 

The severity of the disease varied from mild to severe. The most risk factors for mortality were older age, comorbidity diseases, severity of the illness and leukopenia and lymphopenia. The mortality rate was higher than the normal population. The renal side effects such as acute kidney injury and graft rejection were observed in some studies. The studies mostly recommended reporting more kidney transplant cases infected with COVID-19 and screening for the identification of asymptomatic transfers, educating patients about observing hand hygiene, long term follow-up, close monitoring and providing a single therapeutic guideline for these patients. Hydroxychloroquine was the first choice from antivirals and the azithromycin was used as antibiotic for these patients. The examination of the results of studies showed that no single approach is currently available to the immunosuppressant management of transplant patients infected with SARS-CoV-2. Therefore, different opinions about immunosuppressant agents were collected and summarized, including discontinuation of the immunosuppressant, adjustment of the immunosuppressant (dose and type of drug), not noticing a change in medication and the use of the immunosuppressants in the second phase of the disease (table 1). In the following, we explained the demographic characteristics, clinical signs, laboratory and par clinical examinations, clinical course, treatment, and outcome of a kidney transplant infected with COVID-19.

**Table 1:** The summery of articles based on consumption immunosuppression

**NO.**	**Authors**	**Location**	**Samples**	**Type of Diseases**	**Risk Factors**	**Drugs**	**Kidney damage (%)**	**Mortality Rate (%)**	**Results**	**Recommendation**
1	Alberic F et al,.(7)	Italy	20	Mi & Mo	Comorbidities	HQC, Az, Ant	-	25	Modification (Immunosuppression withdraw and start with methylprednisolone IV)	-
2	Bartiromo M et al,.(8)	Italy	1	S	-	HQC, Ant	0	0	Modification (Initial dose reduction)	Need for therapeutic guidelines in recipients
3	Fontana F et al,.(9)	Italy	1	S	Comorbidities	HQC	0	0	Modification (A single Immunosuppressant)	Need for therapeutic guidelines in recipients
4	Bossini N et al,.(10)	Italy	53	Mi to S	Lymphopnia, higher D-dimer, lack CRP, dyspnea	HQC, Az, Ant	-	7	Modification (mild disease: reduction and severe: Immunosuppression withdrawn and start with methylprednisolone )	Need for therapeutic guidelines in recipients
5	Lauterio A et al,.(11)	Italy	1	S	-	HQC, LPV/r	0	1	Modification	Detection of interaction between immunosuppressants and new antiviral drugs
6	Gandolfini I et al,.(12)	Italy	2	Mo & S	-	HQC, Ant	0	50	Immunosuppression withdraw	-
7	Mella A et al,.(13)	Italy	6	Mo & S	-	HQC, interleukin-6 receptor inhibitor	0	68	Modification (changes according to the patient's condition)	Clinical trials on immunosuppressant effects
8	Maritati F et al,.(14)	Italy	5	Mo & S	-	HQC	0	0	Modification (some withdraw and a single low‐dose)	Detection time of immunosuppressant changes
9	Bussalino E et al,.(15)	Italy	1	Mo	-	HQC, Ant	0	0	Maintaining standard immunosuppressive therapy	Sharing of reports
10	Seminari E et al,.(16)	Italy	1	Mi	-	LPV/r	0	0	Unchanged of the routine immunosuppression.	-
Sub-total		10 (21.3)	91 (11.2)							
11	Zhang H et al,.(17)	China	5	Mi	-	Ant	0	0	Modification (Immunosuppression withdraw and start with methylprednisolone IV)	Sharing of reports
12	Zhu L et al,.(4)	China	10	Mi to S	-	interferon a-2b	0	10	Modification (Immunosuppression withdraw and start with low-dose methylprednisolone)	Sharing of reports
13	Dong C et al,.(18)	China	1	Mo	Elder age	Ant	0	0	Modification (Immunosuppression reduction)	Further study on the antiviral effects on immunosuppressants
14	Wang J et al,.(19)	China	1	S	-	HQC, Ant	0	0	Severe case without discontinuing or reducing immunosuppressant	Screening(education, identification and follow up)
15	Zhang H et al,.(20)	China	27	Mi to S	-	Ant,	0	14.81	Reduction or stopping	Further study on the proportion of immunosuppressants
16	Man Z et al,.(21)	China	1	S	-	interferon a-2b inhalation	0	0	Modification (Immunosuppression withdraw and start with methylprednisolone IV)	Further studies on the immunosuppressive therapy
17	Chen S et al,.(22)	China	1	Mo	Elder age, comorbidities	IVIG	0	0	Modification (Immunosuppression reduction/withdraw and start with low dose methylprednisolone)	More studies for treatment protocol
18	Zhu L et al,.(3)	China	1	S	-	Supportive treatment	0	0	Modification (Immunosuppression withdraw and start with methylprednisolone IV)	Sharing of reports
19	Cheng D et al,.(23)	China	2	S	-	LPV/r	0	0	Modification (Immunosuppression withdraw or reduction and start with low-dose methylprednisolone)	Impact of immunosuppressants on clinical manifestations, severity and outcome
20	Ning L et al,.(24)	China	1	Mi	-	HQC, Az, Ant	0	0	Unchanged of the routine immunosuppression.	Further studies on CD3, CD4, and CD8 levels
Sub-total		10 (21.3)	50(6.1)							
21	Akalin E et al,.(25)	USA	36	Mi to S	-	HQC, Az	0	28	Modification (reduction doses of immunosuppressive agents)	Long term follow up
22	Cravedi P et al,.(26)	USA	144	Mi to S	Elder age, lymphocytopenia,higher LDH,IL6 ,procalcitonin	HQC, Az	52	32	There was no significant association between immunosuppression withdrawal and mortality.	Close monitoring
23	Columbia University Kidney Transplant Program(27)	USA	15	Mi to S		HQC, Az	-	13	Modification (immunosuppression reduction)	Future studies on evaluation of graft function and rejection risk
24	Nair V et al,.(28)	USA	10	Mi to S		HQC, Ant	50	30	Unchanged of the routine immunosuppression.	Comparison of COVID-19 outcomes between transplants and non-transplants
25	Stephanie GY et al,.(29)	USA	12	Mi to S	-	HQC, Ant	0	4.8	Modification (reducing or holding of MMF)	Comparison with large groups of non-transplants
26	Oltean M et al,.(30)	USA	204	Mi to S	Elder age	HQC, Az, Ant	0.5	21.2	Modification (holding of calcineurin inhibitors and antimetabolite during the inpatient)	Close monitoring
27	Chaudhry ZS et al,.(31)	USA	38	Mi to S	Elder age,clinical severity	HQC, Ant	10	22.8	Modification (immunosuppression reduction)	Long term follow up
28	Pereira MR et al,.(32)	USA	46	Mi to S	Elder age, comorbidities	HQC, Az	0	24	Modification (decreasing or stopping of antimetabolite drugs)	Long term follow up and close monitoring
29	Bush R et al,.(33)	USA	1	Mo	-	HQC	0	0	Low-dose maintenance immunosuppressive therapy	Long term follow up
30	Chen TY et al,.(34)	USA	30	Mi to S	-	-	23	20	Modification	Screening(education, identification and follow up)
Sub-total		10 (21.3)	536 (65.8)							
31	Akdur A et al,.(35)	Turkey	1	Mi	-	HQC, Az, Ant	0	0	With no aggressive changes in immunosuppressive doses unless necessary	Further studies on effects and interaction of antiviral drugs
32	Arpali E et al,.(36)	Turkey	1	Mo	Elder age, comorbidities	-	0	0	Modification (immunosuppression reduction)	Long term follow up
33	Demir E et al,.(37)	Turkey	40	Mo & S	Clinical severity	HQC, LPV/r, plasmapheresis, IVIG	0	12	Modification (immunosuppression reduction)	Evaluation of T-cell number, function, and cytokine profile
34	Dirim AB et al,.(38)	Turkey	1	Mo	Comorbidities	LPV/r	0	0	Modification (Mycophenolate mofetil: stopped and tacrolimus dose: reduction).	Reporting of unsuccessful case treatments
Sub-total		4 (8.5)	43(5.3)							
35	Abrishami A et al,.(39)	Iran	12	Mo	-	HQC, LPV/r	-	66	Modification ( immunosuppressant dose reduction)	Further studies on drug interactions with immunosuppressive therapy
36	Ghaffari Rahbar M et al,.(40)	Iran	19	Mi to S	diabetes, changes of tests	HQC, LPV/r	5.6	47.7	Modification (immunosuppression reduction)	Screening(education, identification and follow up)
37	Namazee N et al,.(41)	Iran	1	S		HQC, calcineurin inhibitors	-	100	Modification (immunosuppression reduction)	Evaluation of type and dose of immunosuppressants on severity
Sub-total		3 (6.4)	32(3.9)							
38	Hoek RAS et al,.(42)	The Netherlands	15	Mi to S	Comorbidities, Clinical severity	HQC & Az	-	22	Unchanged of the routine immunosuppressionin 75% patients.	-
39	Meziyerh S et al,.(43)	The Netherlands	1	S	-	HQC & azithro HQC & Az mycin HQC, Az	0	0	Modification (Immunosuppression withdraw and continue with methylprednisolone)	Sharing of reports
Sub-total		2 (4.3)	16 (2)				0	22		
40	Guillen E et al,.(44)	Spain	1	Mo	Comorbidities	HQC, Az, Ant	0	0	Modification (tacrolimus withdraw)	Drug interaction between immunosuppressants and anti-viral drugs
41	Rodriguez-Cubillo B et al,.(45)	Spain	29	S	-	-	0	20.6	Modification (mycophenolate and/or rapamycin withdraw and the dose of calcineurin inhibitors or cyclosporin at low doses)	Long term follow up
Sub-total		2 (4.3)	30(3.7)					20.6		
42	Banerjee D et al,.(46)	UK	7	Mi to S	Changes of tests	-	0	14	Modification (immunosuppression reduction)	Close monitoring
Sub-total		1 (2.1)	7 (0.9)				0	14		
43	Shingare A et al,.(47)	India	2	S	Comorbidities, lower dose of anti-thymocyte globulin (ATG)	-	-	-	Modification ( immunosuppressant dose reduction)	Long term follow up
Sub-total		1 (2.1)	2 (0.2)				-	-0		
44	Machado DJB et al,.(48)	Brazil	1	Mo	-	-	-	-	Modification (immunosuppression reduction)	Drug interaction between immunosuppressants and anti-viral drugs
Sub-total		1 (2.1)	1 (0.1)				-	-		
45	Thammathiwat T et al,.(49)	Thailand	1	S	Elder age	-	-	0	Modification (immunosuppression reduction)	Sharing of reports
Sub-total		1 (2.1)	1 (0.1)				-	0		
46	Marx D et al,.(50)	France	1	Mi	Comorbidities		10	0	Modification (MMF: discontinued and start with Low‐dose cyclosporine)	Screening(education, identification and follow up)
Sub-total		1 (2.1)	1 (0.1)				10	0		
47	Silva F et al,.(51)	Portugal	5	Mi, & Mo	Comorbidities, elder age		0	0	Modification (immunosuppression reduction or withdraw)	-
Sub-total		1 (2.1)	5 (0.6)				0	0		

Mi: Mild; Mo: Moderate; S: Severe; HQC: Hydroxychloroquine; Az: Azithromycine; Ant: Antiviral; , IVIG: Intravenous immune globulin; LPV/r: lopinavir/ritonavir

**Fig 2:**
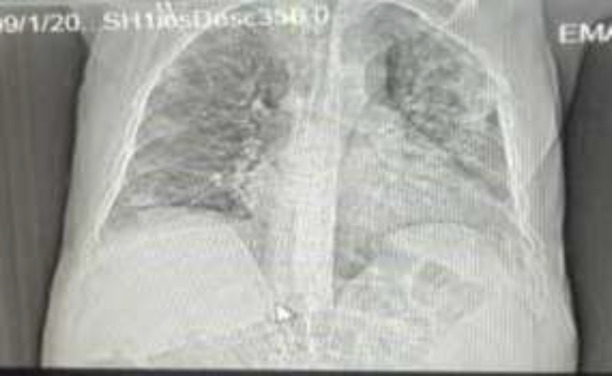
Chest x-ray of the patient with COVID-19 infection

**Patient information:** The patient was a 67-year-old Muslim-Shia male who underwent a kidney transplant 11 years ago. He was self-employed. Five days before hospitalization, he had the symptoms of anorexia, weakness, and lethargy. After that, he started to cough. It was dry and later turned into a cough with sputum. He was first diagnosed with a common cold, but after taking chest x-ray he was hospitalized for COVID-19 (figure 2) with the diagnosis of viral pneumonia on 11.4.2020, in Imam Reza Medical Training Center (one of the reference hospitals in the northwestern region of Iran). 

The patient is a resident of Tabriz, married and has two children. His past medical history was hypertension started 16 years ago, diabetes 10 years ago, and kidney transplantation 11 years ago. The drug history was insulin for diabetes, antihypertensive medicines and mofetil, mycophenolate, prednisolone, and cyclosporine for kidney transplantation. This patient had been recorded at the Organ Transplantation Registry of North-west Iran. He was on home quarantine for the past two months without any hospitalization or travel but was in direct contact with a family member with cold symptoms about 20 days before hospitalization, and had runny nose, sneezing and dry cough.

**Clinical findings:** At the time of admission to the hospital, the patient was conscious and oriented, but lethargic. The assessment of the patient showed tachycardia and crackles in the lungs with Sao2=90 %. Clinical signs, laboratory results, and pharmacotherapy of the patient with COVID-19 infection from the day of admission until discharge have been shown in table 2.

**Diagnostic assessment:** After hospital admission, PCR and other blood tests were carried out for him, in which test results with vital and clinical signs were shown in table 2 from the day 1 of admission until the day of discharge. Pulmonary CT scan and Real-time PCR were reported to be positive for COVID-19. In blood tests, leukocytes were in at least normal range, but lymphocytopenia has not been observed, and inflammatory markers such as ESR and CRP increased. Urea, creatinine, AST, ALT, ALP, LDH, and CPK levels were high and Na, BS, and PaO2 levels were low. In this case, there was bilateral lung involvement, respiratory distress and decreased oxygen saturation.

**Table 2:** Clinical signs laboratory resultsandpharmacotherapy of the patient with COVID-19 infection from the day of admission until discharge

**2020.4.17**	**2020.4.16**	**2020.4.15**	**2020.4.14**	**2020.4.13**	**2020.4.12**	**2020.4.11**	**Variables**
36.6	36.6	37	36.4	36.1	36.6	36.6	BT (C)
96	110	100	110	98	100	115	PR
20	19	20	21	26	32	32	RR
110/70	110/70	110/70	125/85	120/80	110/70	130/80	BP (mmHg)
97 95 96	97 95 93 92	93 92	91 92	93 92 90 91	90 92 93 91	90 92 93	SaO2 % without O2
5300	4500	4500	3700	3700	3700	4500	WBC (*1000/mm2)
		-	-	-	-	12.5%	Lymphosite
16.9	16.9	-	-	-	-	17.1	Hb (g/dL)
85	228	-	-	-	-	161	Plt (*1000/mm2)
62/92	-	-	-	-	-	75/98	ESR
-	2+	2+	2+	2+	2+	2+	CRP
110 136	160 136 133 170	128 135 190	73 203 240	152 143	65 135 160	60	BS (mg/dl)
61	73	91	93	92	86	94	Urea (mg/dl)
1.05	1.60	1.91	2.29	1.95	1.96	2.20	Cr (mg/dl)
142	134	130	133	127	125	120	Na (mEq/l)
5	4.4	4	4.5	4.3	-	4.7	K (mEq/l)
2.3	-	-	-	-	-	1.2	Mg (mg/dl)
1.37	-	-	1.4	-	-	1.4	Ca (mmol/L)
	-	-	2.4	-	-	-	P (mg/dl)
151	-	-	171	-	-	122	AST (IU/L)
70	-	-	44	-	-	63	ALT (IU/L)
166	-	-	234	-	-	500	ALP (IU/L)
-	751	-	-	-	639	-	LDH (IU/L)
-	63	-	-	-	104	-	CPK (IU/L)
7.39	7.35	-	7.35	7.37	7.38	7.42	PH
28.9	25.4	-	29.3	25.5	24	24.2	PCO2 (mmHg)
40	40	-	75.6	68	63	59	PO2 (mmHg)
16	13.8	-	16	14.9	14.9	15.7	HCO3 (mol/L)
95	95	93	91	93	90	92	SO2
Tab Hydroxychloroquine- Tab Kaletra- Cap Omeprazole Amp Heparin- Ser Normal Saline- Tab Allopurinol Tab Nitrocontin - Insulin Lantus- Insulin Nor rapid Tab Cellcept- Tab Atorvastatin- Tab ASA- Tab Prednisolone- Tab Amlodipine Tab Digoxin- Amp Mgso4 20%	Tab Hydroxychloroquine- Tab Kaletra- Cap Omeprazole- Amp Heparin- Serum Normal Saline- Tab Allopurinol Tab Nitrocontin - Insulin Lantus- Insulin Nor rapid Tab Cellcept- Tab Atorvastatin Tab ASA- Tab Prednisolone- Tab Amlodipine Tab Digoxin- Amp Mgso4 20%	Tab Hydroxychloroquine- Tab Kaletra- Cap Omeprazole Amp Heparin- Serum Normal Saline- Tab Allopurinol Tab Nitrocontin - Insulin Lantus- Insulin Nor rapid Tab Acetaminophen- Tab Cellcept- Tab Atorvastatin- Tab ASA- Tab Prednisolone Tab Amlodipine- Tab Digoxin- Amp Mgso4 20%	Tab Hydroxychloroquine- Tab Kaletra- Cap Omeprazole- Amp Heparin- Serum Half Saline- Tab Allopurinol Tab Nitrocontin - Insulin Lantus- Insulin Nor rapid- Tab Acetaminophen- Tab Cellcept- Tab Atorvastatin- Tab ASA- Tab Prednisolone- Tab Amlodipine- Tab Digoxin- Amp Mgso4 20%	Tab Hydroxychloroquine- Tab Kaletra- Omeprazole- Amp Heparin- Serum Half Saline- Tab Allopurinol- Tab Nitrocontin - Insulin Lantus- Insulin Nor rapid- Tab Acetaminophen Tab Cellcept- Tab Atorvastatin- Tab ASA	Tab Hydroxychloroquine- Tab Kaletra		Drugs

BT: Body temperature, PR: Pulse rate, RR: Respiratory rate, BP: Blood pressure, SaO2: Oxygen saturation, WBC: White blood cell, HB: Hemoglobin, Het: Hematocrit, PLT: Platelet

**Therapeutic interventions:** Semi fowler position, oxygen therapy with canola, serum therapy, and diabetic diet have been prescribed for him as follows:

Hydroxychloroquine tab. 200 mg twice-daily; Kaletra tab. 200MG /50MG per day; Omeprazole cap. 20 mg per day; Cimetidine amp. 200 mg QID; Amlodipine tab. 5 mg BID; Digoxin tab. 0.25 mg per day; Atorvastatin tab. 10 mg per day; Insulin injection based on the blood sugar every 6 hours; Heparin injection. 5000 units TDS; Magnesium infusion. 600 mg TDS; Aspirin tab. 80 mg per day; Allopurinol tab. 300mg per day as well as 5 mg of Prednisolone tab daily, and 500 mg of CellCept tab BID were taken during 5 hospitalization days. It should be noted that a corticosteroid regimen (prednisolone and cellcept) was similar to the treatment done before hospital admission.

**Follow-up and outcomes:** The patient was discharged on 17-4-2020 in a stable condition without respiratory distress. He was recommended to be at home quarantined for 14 days by observing a 2-meter distance from his wife and daughter. The PCR-Real Time test of his wife and daughter was negative. After a month of follow-up, the general condition of the patient was satisfactory without any symptoms of cough or dyspnea. The PCR-Real Time was negative. The level of Cr was 1.08 mg/dl. 

## Discussion

Following the outbreak of acute respiratory syndrome (SARS) and Middle East respiratory syndrome (MERS), transplant patients were at an increased risk for infection, mortality, and organ rejection due to the utilization of immunosuppressive agents (6). Another important clinical issue is the following treatment of immunosuppressive drugs in organ transplant recipients over of the viral infections resulting in epidemics and pandemics such as COVID-19 (8, 23). Long-term immunosuppressive drugs are essential in organ transplantation for the prevention of rejection (51). Therefore, it is necessary to maintain the integrity of the immunosuppressive therapy used routinely in posttransplant patients (8). Now, if another infection like COVID-19 is added, the careful balancing of an immunosuppressant is more difficult (6). Therefore, we surveyed the opinions of the authors in kidney transplant infected with COVID-19 after the diagnosis of COVID-19 and hospital admission for a kidney transplant patient. He was diagnosed early with atypical symptoms despite having the effects of multiple risk factors for COVID-19, including old age, male gender, pulmonary involvement, elevated inflammatory markers, history of diabetes, and history of hypertension. After an organ transplant, immunosuppressant agents are prescribed to organ transplant recipients to prevent and treat rejection (26, 46). The results of the literature review showed that the transplant patients with COVID- 19 should be hospitalized even with mild forms of disease (25). According to the results of the studies in the early stage of the COVID- 19 for kidney recipients, the severity of the disease is higher and the length of the disease is longer, due to the occurrence of the asymptomatic symptoms (22, 41). More transplant patients receive a triple combination regimen consisting of tacrolimus (TAC), mycophenolate mofetil (MMF), and prednisone (Pred) after transplantation (10, 17, 50). However, there were different opinions about the use of immunosuppressants including withdrawal (13), modification (25), and or unchanged (17, 24) of immunosuppressive therapy. Withdrawal or reduction of immunosuppressant agents in kidney transplants infected with COVID-19 may limit adverse events and prevent sustained viral presence and spreading in receptions (11, 47). 

Otherwise, stopping or decreasing these agents may lead to destructive outcomes such as allograft rejection or relapse in inflammatory conditions (17, 28). Patients infected with COVID-19 sometimes enter into a severe inflammatory phase where immunosuppressant agents are important in reducing inflammation and preventing organ damage in the body (21, 29, 31). In a study it was mentioned that Hydroxychloroquine was effective in mild cases. Azithromycin could be added to the treatment regime if the disease progresses and in severe cases, the use of antivirals is also recommended (35). With regard to treatment protocols should be according to the stage and severity of the disease. Because of the interactions between immunosuppressant and other drugs, it is necessary to check the concentration of the drugs (1). In review studies, further experience has been suggested over the dose and time adjustment, and interactions with other drugs and antivirals (37, 40, 44, 50). Different immunosuppressive therapies were proposed due to encounter-based experiences with other viral infections in kidney transplant recipients (41). Nevertheless, it seems that the immunosuppressants alone could be a therapeutic choice in kidney transplants infected with COVID-19 and drug regulatory decision-making should be considered on a case-to-case basis and consider patients' condition such as the clinical symptoms (fever-respiratory symptoms), laboratory results (leukopenia-lymphocytopenia), and chest CT scan results (lung involvement) (12, 14, 41, 47); however, the diagnosis of COVID- 19 among these patients is a challenge for physicians. To get the best result, it is better to compare the symptoms, severity and outcomes of the COVID- 19 between transplant patients with the normal population (26). Here we are going to present a kidney transplant reception infected with COVID-19 and was treated based on clinical signs without changes in immunosuppressive therapy. 

The presented patient firstly is diagnosed as having a common cold. Various studies have shown that COVID-19 leads to fever, dry cough, and shortness of breath, myalgia, sore throat, and fatigue (1). In a systematic review conducted by Lovato A et al., the most common symptoms of COVID- 19 were fever, cough, fatigue, and shortness of breath, myalgia, sore throat, headache, nausea, vomiting, and diarrhea, respectively (53). The patients under immunosuppressive drugs may not show the classic clinical symptoms; therefore, introducing the common and non-common clinical symptoms of the disease via the Ministry of Health notifications and the media news could be helpful in the early detection of the disease.

In this patient, the chest CT scan and RT-PCR were positive for COVID-19. The initial diagnosis of COVID-19 infection is based on clinical signs; however, the results of lab tests, chest CT scan, and RT-PCR are very effective in making a definitive diagnosis of the disease (27, 37). These findings are important in the diagnosis, treatment, management, and follow-up of the disease (31). 

In the study of Lovato A et al., the involvement of chest x-ray, leukopenia, and lymphocytopenia were reported in 80%, 30% and 77% of patients, respectively (1). In another study, lymphocytopenia, leukopenia, increased CRP, and lactic dehydrogenase were reported in 64.5%, 29.4%, 44.3%, and 28.3% of cases, respectively (54). As mentioned, the interesting point in this study was the improvement of the patient despite having different risk factors for the disease. The studies showed that old age, cardiovascular and respiratory problems, diabetes, hypertension, obesity, smoking, and cancer are risk factors in terms of increasing the severity of the disease, the length of in-hospital stay, morbidity and mortality for COVID-19 (55). Lymphopenia, which is caused by the use of immunosuppressive drugs in the body, could be a risk factor for high mortality in COVID-19 outbreaks (25).

In a study led by Elens L et al., transplant recipients were more likely to develop COVID-19, especially severe form, due to the chronic use of immunosuppressive drugs and immune system suppression (56). In contrast, immunosuppressive drugs with an appropriate management in the transplant patients, is helpful, but longer follow-up is needed for these patients (44). The organ transplant recipients infected with COVID-19 may experience different clinical signs, clinical courses, treatment and even laboratory results due to the immunosuppressants. The immunosuppressant regulation could be considered based on different conditions such as clinical symptoms, laboratory results, paraclinical examinations. The clinical trial studies are recommended for better results. The main limitation of this case study was the availability in patient after discharge for further follow-ups. The strengths were recognition and study of this case by the Organ Transplant Registry team. 

Nevertheless, this is a single case report and the results of this study could not extrapolate as a medical recommendation for transplant patients infected with COVID-19, but the current results in combination with other studies’ results could be used for providing a treatment protocol for these patients.

**Abbreviations:**

COVID-19: Corona Virus Disease 2019, SARS: Acute Respiratory Syndrome, MERS: Middle East Respiratory Syndrome, CRP: C-reactive protein, COPD: Chronic obstructive pulmonary disease, CT: Computed Tomography, RT-PCR: Real-time polymerase chain reaction, AST: Aspartate aminotransferase, ALT: Alanine aminotransferase, ALP: Alkaline Phosphatase, LDH: Lactate dehydrogenase, CPK: Creatine phosphokinase, Tab: Tablet, Cap: Capsule.

## References

[1] Pan L, Mu M, Yang P (2020). Clinical characteristics of COVID-19 patients with digestive symptoms in Hubei, China: a descriptive, cross-sectional, multicenter study. Am J Gastroenterol.

[2] Ozma MA, Maroufi P, Khodadadi E (2020). Clinical manifestation, diagnosis, prevention and control of SARS-CoV-2 (COVID-19) during the outbreak period. Infez Med.

[3] Zhu L, Xu X, Ma K (2020). Successful recovery of COVID‐19 pneumonia in a renal transplant recipient with long‐term immunosuppression. Am J Transplant.

[4] Zhu L, Gong N, Liu B (2020). Coronavirus disease 2019 pneumonia in immunosuppressed renal transplant recipients: a summary of 10 confirmed cases in Wuhan, China. Eur Urol.

[5] Elens L, Langman LJ, Hesselink DA (2020). Pharmacologic treatment of transplant recipients infected with SARS-CoV-2: Considerations Regarding Therapeutic Drug Monitoring and Drug–Drug Interactions. Ther Drug Monit.

[6] Boyarsky BJ, Po‐Yu Chiang T, Werbel WA (2020). Early impact of COVID‐19 on transplant center practices and policies in the United States. Am J Transplant.

[7] Alberici F, Delbarba E, Manenti C (2020). Management of patients on dialysis and with kidney transplant during SARS-COV-2 (COVID-19) pandemic in Brescia, Italy. Kidney Int Rep.

[8] Bartiromo M, Borchi B, Botta A (2020). Threatening drug‐drug interaction in a kidney transplant patient with Coronavirus Disease 2019 (COVID‐19). Transpl Infect Dis.

[9] Fontana F, Alfano G, Mori G (2020). Covid‐19 pneumonia in a kidney transplant recipient successfully treated with Tocilizumab and Hydroxychloroquine. Am J Transplant.

[10] Bossini N, Alberici F, Delbarba E (2020). Kidney transplant patients with SARS‐CoV‐2 infection: the brescia renal COVID task force experience. Am J Transplant.

[11] Lauterio A, Valsecchi M, Santambrogio S (2020). Successful recovery from severe COVID‐19 pneumonia after kidney transplantation: The interplay between immunosuppression and novel therapy including tocilizumab. Transplant Infect Dis.

[12] Gandolfini I, Delsante M, Fiaccadori E (2020). COVID‐19 in kidney transplant recipients. Am J Transplantation.

[13] Mella A, Mingozzi S, Gallo E (2020). Case series of six kidney transplanted patients with COVID‐19 pneumonia treated with tocilizumab. Transplant Infect Dis.

[14] Maritati F, Cerutti E, Zuccatosta L (2020). SARS‐CoV‐2 infection in kidney transplant recipients: experience of the italian marche region. Transplant Infect Dis.

[15] Bussalino E, De Maria A, Russo R, Paoletti E (2020). Immunosuppressive therapy maintenance in a kidney transplant recipient with SARS‐CoV‐2 pneumonia: A case report. Am J Transplant.

[16] Seminari E, Colaneri M, Sambo M (2020). SARS Cov‐2 infection in a renal‐transplanted patient: A case report. Am J Transplant.

[17] Zhang H, Chen Y, Yuan Q (2020). Identification of kidney transplant recipients with coronavirus disease 2019. Eur Urol.

[18] Chen D, Yang B, Zhang Y (2020). Withdrawing mycophenolate mofetil in treating a young kidney transplant recipient with COVID-19: A case report. Medicine.

[19] Wang J, Li X, Cao G (2020). COVID-19 in a kidney transplant patient. Eur Urol.

[20] Zhang H, Dai H, Xie X (2020). Solid Organ Transplantation During the COVID-19 Pandemic. Front Immunol.

[21] Zhang M, Zhang J, Shi H, Liu B, Zeng F (2020). Viral shedding prolongation in a kidney transplant patient with COVID‐19 pneumonia. Am J Transplant.

[22] Chen S, Yin Q, Shi H (2020). A familial cluster, including a kidney transplant recipient, of Coronavirus Disease 2019 (COVID‐19) in Wuhan, China. Am J Transplant.

[23] Cheng D, Wen J, Liu Z, Lv T, Chen J (2020). Coronavirus disease 2019 in renal transplant recipients: report of two cases. Transplant Infect Dis.

[24] Ning L, Liu L, Li W (2020). Novel coronavirus (SARS‐CoV‐2) infection in a renal transplant recipient: case report. Am J Transplant.

[25] Akalin E, Azzi Y, Bartash R (2020). Covid-19 and kidney transplantation. N Engl J Med.

[26] Cravedi P, Mothi SS, Azzi Y (2020). COVID‐19 and kidney transplantation: results from the TANGO International Transplant Consortium. Am J Transplant.

[27] Columbia University Kidney Transplant Program (2020). Early description of coronavirus 2019 disease in kidney transplant recipients in New York. J Am Soc Nephrol.

[28] Nair V, Jandovitz N, Hirsch JS (2020). COVID‐19 in kidney transplant recipients. Am J Transplant.

[29] Stephanie GY, Rogers AW, Saharia A (2020). Early experience with COVID-19 and solid organ transplantation at a US high-volume transplant center. Transplantation.

[30] Oltean M, Søfteland JM, Bagge J (2020). Covid-19 in kidney transplant recipients: a systematic review of the case series available three months into the pandemic. Infectious Dis.

[31] Chaudhry ZS, Williams JD, Vahia A (2020). Clinical characteristics and outcomes of COVID‐19 in solid organ transplant recipients: a case‐control study. Am J Transplant.

[32] Pereira MR, Mohan S, Cohen DJ (2020). COVID‐19 in solid organ transplant recipients: Initial report from the US epicenter. Am J Transplant.

[33] Bush R, Johns F, Acharya R, Upadhyay K (2020). Mild COVID‐19 in a pediatric renal transplant recipient. Am J Transplant.

[34] Chen TY, Farghaly S, Cham S (2020). COVID‐19 pneumonia in kidney transplant recipients: Focus on immunosuppression management. Transplant Infect Dis.

[35] Akdur A, Karakaya E, Ayvazoglu Soy EH (2020). Coronavirus Disease (COVID-19) in kidney and liver transplant patients: a single-center experience. Exp Clin Transplant.

[36] Arpali E, Akyollu B, Yelken B (2020). Case report: A kidney transplant patient with mild COVID‐19. Transpl Infect Dis.

[37] Demir E, Uyar M, Parmaksiz E (2020). COVID‐19 in kidney transplant recipients: A multicenter experience in Istanbul. Transplant Infect Dis.

[38] Dirim AB, Demir E, Ucar AR (2020). Fatal SARS-CoV-2 infection in a renal transplant recipient. CEN Case Rep.

[39] Abrishami A, Samavat S, Behnam B (2020). Clinical course, imaging features, and outcomes of COVID-19 in kidney transplant recipients. Eur Urol.

[40] Ghaffari Rahbar M, Nafar M, Khoshdel A (2020). Low rate of COVID‐19 pneumonia in kidney transplant recipients‐a battle between infection and immune response?. Transpl Infect Dis.

[41] Namazee N, Mahmoudi H, Afzal P, Ghaffari S (2020). Novel Corona Virus 2019 pneumonia in a kidney transplant recipient. Am J Transplant.

[42] Hoek RA, Manintveld OC, Betjes MG (2020). Covid‐19 in solid organ transplant recipients: A single center experience. Transpl Int.

[43] Meziyerh S, Zwart TC, van Etten RW (2020). Severe COVID‐19 in a renal transplant recipient: A focus on pharmacokinetics. Am J Transplant.

[44] Guillen E, Pineiro GJ, Revuelta I (2020). Case report of COVID‐19 in a kidney transplant recipient: does immunosuppression alter the clinical presentation?. Am J Transplant.

[45] Rodriguez‐Cubillo B, Moreno de la Higuera MA, Lucena R (2020). Should cyclosporine be useful in renal transplant recipients affected by SARS‐CoV‐2?. Am J Transplant.

[46] Banerjee D, Popoola J, Shah S (2020). COVID-19 infection in kidney transplant recipients. Kidney Int.

[47] Shingare A, Bahadur MM, Raina S (2020). COVID‐19 in recent kidney transplant recipients. Am J Transplant.

[48] de Barros Machado DJ, Ianhez LE (2020). COVID‐19 pneumonia in kidney transplant recipients‐where we are?. Transpl Infect Dis.

[49] Thammathiwat T, Tungsanga S, Tiankanon K (2020). A case of successful treatment of severe COVID‐19 pneumonia with favipiravir and tocilizumab in post‐kidney transplant recipient. Transpl Infect Dis.

[50] Marx D, Moulin B, Fafi‐Kremer S (2020). First case of COVID‐19 in a kidney transplant recipient treated with belatacept. Am J Transplant.

[51] Silva F, Cipriano A, Cruz H (2021). SARS‐CoV‐2 infection in kidney transplant recipients. Transplant Infect Dis.

